# The relationship between sleep disorders and psychotic-like symptoms in the general population

**DOI:** 10.1192/j.eurpsy.2021.451

**Published:** 2021-08-13

**Authors:** L. Fusar-Poli, T. Surace, A. Aguglia, E. Aguglia

**Affiliations:** 1 Department Of Clinical And Experimental Medicine, University of Catania, Crema (CR), Italy; 2 Department Of Neuroscience, Rehabilitation, Ophthalmology, Genetics Maternal And Child Health, University of Genoa, Genoa, Italy; 3 Department Of Clinical And Experimental Medicine, University of Catania, Catania, Italy

**Keywords:** psychotic-like symptoms, sleep disorders, General population, Insomnia

## Abstract

**Introduction:**

Abnormalities of sleep patterns are common in people with psychiatric disorders and often represent a source of distress, worsening the outcome. However, little is knwon about the relationship between psychotic-like symptoms and sleep disorders in the general population.

**Objectives:**

1. Whether there is a relationship between sleep disorders and psychotic-like experiences in a sample of individuals belonging to the general population. 2. Which sleep disorders are more commonly associated with psychotic-like experiences.

**Methods:**

A web survey was spread thorugh social networks. We administered the SLEEP-50 to investigate the presence of sleep disorders and the Community Assessment of Psychic Experience (CAPE) for psychotic-like symptoms. Moreover, socio-demographic characteristics of participants were collected.

**Results:**

The web-survey was completed by 824 participants. Six people refused to give consent and 95 were excluded because they declared to suffer from psychiatric disorder sor other medical conditions potentially infleuncing on sleep. Therefore, 729 subjects were included in the analysis. Pearson correlation coefficients showed strong correlations between the scale regarding SLEEP-50 “All sleep disorders” scale and CAPE Total and Depressive scales (r = 0.52, p < 0.001). A moderate correlation was found between “All sleep disorders” and CAPE Negative (r = 0.49) and Positive (r = 0.32) scales. Correlations with specific SLEEP-50 subscales were also found.

**Conclusions:**

There seems to be a strong relationship between psychotic-like symptoms and sleep problems in the general population. Our findings might indicate that some sleep abnormalities may represent earlier symptoms of a psychiatric condition and need to be always monitored even in the non-psychiatric population.

**Disclosure:**

No significant relationships.

